# Magnetic resonance imaging features of post-COVID-19 regional and invasive sino-nasal mucormycosis

**DOI:** 10.1186/s43055-022-00930-w

**Published:** 2022-11-28

**Authors:** Ahmed Gamil Ibrahim Abd El Megid, Ghada Adel AbdelHamid, Mohamed El-Sayed Abd Elbary, Nesma A. M. Ghonimi, Ahmed I. Elagrody, Marwa Elsayed Abd Elhamed

**Affiliations:** 1 Radiology Department, Faculty of Medicine, Zagazig University, Zagazig City, Sharkia Governorate Egypt; 2 ENT Department, Faculty of Medicine, Zagazig University, Zagazig City, Sharkia Governorate Egypt; 3 Neurology Department, Faculty of Medicine, Zagazig University, Zagazig City, Sharkia Governorate Egypt; 4 Internal Medicine Department, Faculty of Medicine, Zagazig University, Zagazig City, Sharkia Governorate Egypt

**Keywords:** MRI, COVID-19, Mucormycosis

## Abstract

**Background:**

Sino-nasal mucormycosis is an opportunistic, invasive fungal disease which has shown a rising trend in the setting of COVID-19. The objective of this study is to document and analyze demographic data, clinical presentation and MR imaging spectra for early detection and management of post-COVID-19 sino-nasal mucormycosis.

**Results:**

Sixty-two cases of sino-nasal mucormycosis were enrolled in this study; their mean age was 50.65 ± 8.25 years, with significant female predominance. Nine patients (14.5%) had active COVID-19 and 53 (85.5%) were recent COVID-19 cases. Sixty patients have not received COVID-19 vaccine. The mean duration from the initial COVID-19 laboratory confirmation to the detection of sino-nasal mucormycosis was 25.7 +/− 4.6 days. Thirty-five patients (56.5%) were kept in the hospital for COVID management and 4 of them received intensive care unit (ICU) treatment. Twenty-seven patients (43.5%) were treated in home isolation. Corticosteroids were administered in 48 cases (77.4%). Twenty-nine patients (46.8%) had been given oxygen for an average time of 11.2 ± 4.15 days. Diabetes was found in 56 cases (90.3%). The most common clinical symptoms were headache, seen in 52 patients (83.87%). The ethmoid sinus was the most common paranasal sinus involved in our study, seen in 47 cases (75.81%). In 36 cases (58%), multiple sinuses were involved. MRI staging according to the extent of regional involvement. Stage 1 seen in 2 cases (3.23%), stage 2 in 13 cases (20.97%), stage 3 in 35 cases (56.45%) and stage 4 in 12 cases (19.35%).

**Conclusions:**

MRI shows a spectrum of findings in sino-nasal mucormycosis. Imaging plays a major role in staging and assessing the extent of involvement and complications. In light of this, mortality and morbidity can be dramatically decreased with adequate evaluation and therapy.

## Background

The 2019 novel coronavirus disease (COVID-19) is an infectious viral disease caused by the severe acute respiratory syndrome coronavirus 2 (SARS-CoV-2) [1]. COVID-19 is known to cause respiratory symptoms primarily, ranging from mild to severe pneumonia [2]. However, a variety of bacterial and fungal co-infections can be associated with it [3].

In hospitalized and critically ill COVID-19 patients, secondary infections are apparently widespread, with fungal infections being 10 times more likely [4].

Mucormycosis is an opportunistic, invasive fungal disease is brought on by saprophytic fungi of the order Mucorales [5]. Hypoxia, uncontrolled blood sugars by steroids or by diabetic mellitus, and protracted multifaceted immunosuppression are some of the suggested risk factors [5]. Although it can affect different organs, rhino-orbitocerebral mucormycosis is the most prevalent type (ROCM) [6].

Due to the suppressed immune response and impaired glucose homeostasis caused by corticosteroids, they have been recognized as a possible risk factor for mucormycosis infection. Additionally, severe COVID-19 disease has been linked to a hyperferritinemic condition, which raises the possibility of cellular deterioration and more free iron, both of which raise the possibility of mucormycosis [7, 8]. It has also been hypothesized that the rise in post-COVID-19 mucormycosis is related to the widespread use of dietary supplements that contain zinc and iron, two nutrients important for Mucorales. According to Sen et al. [9], ROCM was seen in patients with severe COVID-19 illness, those who were hospitalized to the critical care unit, or those who needed mechanical ventilation [9]. According to Banerjee et al. [10], other factors that may lead to ROCM in COVID-19 patients include misuse of industrial oxygen, as well as reusable oxygen humidifiers [10].

The most frequent initial symptoms include headache, fever, facial edema, facial pain, nasal discharge and nasal ulceration [11]. About 50% of patients experience a foul-smelling, black, necrotic eschar on their palate or nasal mucosa, although this is a sentinel sign of ROCM [10, 12]. Localized vascular thrombosis and tissue infarction are the causes of this necrotic black tissue [10]. The degree of orbital involvement ranges from 66 to 100% [13]. Reduced vision, vitritis, endophthalmitis, ophthalmoplegia, orbital apex syndrome, orbital cellulitis, periorbital edema, chemosis and proptosis are all manifestations of orbital spread [11]. Involvement of the orbital apex or infarction of the optic nerve caused by ophthalmic/central retinal arterial obstruction may result in blindness, a serious complication of ROCM [13]. Direct extension or angioinvasion are two ways that intracranial advancement may take place, typically over a few days [14]. Almost all patients have orbital involvement when intracranial illness is discovered [11]. Hemiparesis, abnormal mentation, cavernous sinus thrombosis, and focal seizures are warning signs of intracranial spread [12].

A four-stage approach was proposed by Honavar SG [15] to assess the anatomical scope and severity of ROCM. Along with clinical symptoms and signs, it also contains the findings from diagnostic tests including imaging and nasal endoscopy. It categorizes ROCM into four stages: stage I, which affects just the nasal mucosa, stage II, which spreads to the paranasal sinuses, stage III, which affects the orbit, and stage IV which involves the central nervous system [15].

MRI is superior to CT in evaluation of sino-nasal mucormycosis due to its improved anatomic and pathologic resolution and enhanced slice orientation. An obvious benefit of MRI over CT scanning is its capacity to reveal cross-sectional anatomy and disease with improved tissue identification and even without the use of intravenous gadolinium-based contrast medium. Contrast injection helps distinguish living from dead necrotic tissue in sinusitis, and it also helps distinguish phlegmon from abscess in the orbit and intracranial extensions. The identification of involvement of the cavernous sinus and perineural spread, which are dangerous consequences of fungal sinusitis, is most accurate with MRI [4].

## Methods

This prospective study was conducted in MRI unit of Radiology department of Zagazig University Hospitals, Egypt, in the period from 1st of November 2021 to end of March 2022. All patients referred from our hospitals with suspected or proved sino-nasal mucormycosis. The approval of this prospective study was obtained from the institutional review board and written consents from all subjects were achieved.

The inclusion criteria were patients with confirmed recent or active COVID-19 infection, with clinical features suggestive of sino-nasal mucormycosis, supported by nasal endoscopic results or imaging was identified as probable sino-nasal mucormycosis and having written informed consent to participate in the research. No gender or age limits.

The exclusion criteria were patients not confirmed to have active or recent COVID-19 infection, patients with unavailable clinical and laboratory data, and patients with previous history of head and neck surgeries.

Reverse transcriptase polymerase chain reaction (RT-PCR) results from a naso-oropharyngeal swab that were documented as positive served as the basis for the COVID-19 diagnosis. Patients were classified as recent COVID-19 if they had recovered from COVID-19 infection three months prior and were currently RT-PCR SARS-CoV2 negative. According to universally accepted description of post-acute COVID-19 syndrome, a three-month time frame was chosen. Patients with SARS-CoV-2 confirmation (by RT-PCR) at presentation were considered to be active COVID-19 cases [16].

A thorough data collection form was developed to gather information from hospital records based on the patient's demographic profile, vaccination status, date of COVID-19 diagnosis, time since the onset of symptoms, clinical characteristics of sino-nasal mucormycosis, co-morbidities, COVID-19 treatment received, nasal endoscopy findings, and microbiological, pathological, and radiological details.

MRI examinations were done using a 1.5 Tesla superconducting magnet (Achieva, Philips Medical System, Best, Netherlands), using a standard head coil.

All subjects underwent Contrast-enhanced MRI (CE-MRI) protocol of the paranasal sinuses, brain, and orbits included axial T1, T2, FLAIR, GRE, DWI, T2 FS, T1 FS post-contrast (3 mm thickness), sagittal T2 and T1 FS post-contrast (3 mm thickness), coronal T2 and T1 FS post-contrast (3 mm thickness) sequences.

At the same time, all of the patients' images were evaluated by two competent radiologists (a competent radiologist is a radiologist who has more than five years of experience in the field of Neuro-imaging). In case of disagreement, a third radiologist with more competence in neuro-imaging was consulted for advice (more than 8 years).

This study demonstrates the MRI features of sino-nasal mucormycosis in post-COVID-19 patients, including the traditional diagnostic signs and illness staging, as well as the pathways of infection transmission. The goal of this study is to acquaint each member of the multidisciplinary team handling these patients with how to interpret the results of an MRI in sino-nasal mucormycosis.

Histopathological evaluation of nasal discharge of all cases was done on potassium hydroxide (KOH) wet mount and further confirmed on culture using lactophenol cotton blue (LPCB) stain. Final diagnosis of mucormycosis was made based on clinical details, imaging findings, and histopathology.

### Statistical analysis of the data

Data were fed to the computer and analyzed using IBM SPSS software package version 20.0*.* (Armonk, NY: IBM Corp). Qualitative data were described using number and percent. Quantitative data were described using range (minimum and maximum), mean, standard deviation, median and interquartile range (IQR).

## Results

**Fig. 1:**
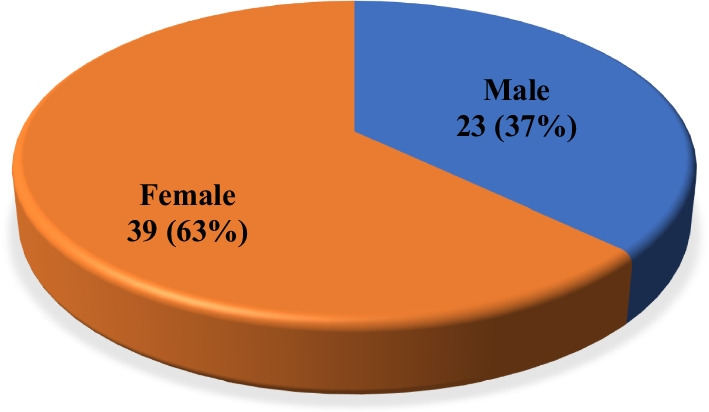
Sex distribution pie for sino-nasal mucormycosis

Sixty-two (62) cases of sino-nasal mucormycosis were enrolled in this study; their mean age was 50.65 ± 8.25 years [range 13–72 years; 23 males (37%), and 39 females (63%)]. The demographic profile is shown in Fig. 1.

Active COVID-19 was seen in 9 patients (14.5%), while recent COVID-19 cases in 53 patients (85.5%). Sixty patients not received COVID-19 vaccine, two were vaccinated; received two doses of vaccine.

The average time between the initial laboratory COVID-19 confirmation and detection of sino-nasal mucormycosis was 25.7 +/− 4.6 days (range 17–39 days).

Forty-eight instances (77.4%) received corticosteroids; 17 received oral (prednisolone/methylprednisolone) and 31 had parenteral (methylprednisolone/dexamethasone) treatment.

Thirty-five patients (56.5%) were hospitalized for COVID management, 4 of them received intensive care unit (ICU) treatment. Twenty-seven patients (43.5%) were treated in home isolation. Corticosteroids were administered in 48 cases (77.4%), 17 received oral (prednisolone/methylprednisolone) and 31 parenteral (methylprednisolone/dexamethasone) treatment. Twenty-nine patients (46.8%) had received oxygen for an average of 11.2 ± 4.15 days.

Pre-existing diabetes was detected in 49 cases (79%), that ranged in duration from 1 to 23 years and newly diagnosed diabetes was seen in 7 cases (13%). Hypertension was the second most prevalent co-morbidity after diabetes, found in 36 cases (58%), followed by chronic renal disease in 8 cases (13%), chronic cardiac disease in 7 cases (11.3%), chronic hepatic disease in 5 cases (8.1%), chronic obstructive pulmonary disease in 5 cases (8.1%), asthma in 4 cases (6.5%), leukemia in 1 case (1.6%) and renal transplant in 1 case (1.6%). Forty-one patients (66.13%) had more than one co-morbidity. No co-morbidity found in 4 cases (6.5%) (Table 1).

**Table 1:** Co-morbidities for the studied group

Comorbidity	*N*	%
Non	4	6.5
DM	56	90.3
Hypertension	36	58.1
Chronic renal disease	8	12.9
Chronic cardiac disease	7	11.3
Chronic hepatic disease	5	8.1
Chronic obstructive lung disease	5	8.1
Asthma	4	6.5
Leukemia	1	1.6
Renal transplant	1	1.6

### Clinical features

The clinical presentation varied. The most common clinical symptoms were headache, seen in 52 patients (83.87%) and cheek swelling in 45 patients (72.58%) and the least was facial palsy in two patients (3.2%). Note that some patients presented with more than one clinical parameter (Table 2).

**Table 2:** Clinical presentation for the studied group

Clinical presentation	*N*	%
Fever	9	14.52
Headache	52	83.87
Nasal blockage	21	33.87
Blood/black stained nasal discharge	34	54.84
Epistaxis	15	24.19
Cheek swelling	45	72.58
Facial pain	39	62.9
Facial swelling	38	61.29
Periocular edema	24	38.71
Eyelids swelling	35	56.45
Black discoloration of the eye lid	17	27.42
Ptosis	32	51.61
Proptosis	16	25.81
Diplopia	7	11.29
Diminution/loss of the vision	6	9.68
Ophthalmoplegia	23	37.1
Facial palsy	2	3.23
Altered mentation	4	6.45
Seizures	4	6.45
Hemiparesis/hemiplegia	3	4.84

### Microbiological examination

All patients' samples with KOH mounts showed broad, aseptate hyphae with wide angles suggestive of Mucor species. Mucor, Candida, and Aspergillus species were present as mixed flora in three patients.

### Sino-nasal involvement

The ethmoid sinus was the most common paranasal sinus involved in our study, seen in 47 cases (75.81%). In 36 cases (58%), multiple sinuses were involved. Pan sinusitis seen in 15 cases (24.19%). The sinuses involved in mucormycosis are detailed in Table 3.

**Table 3:** Sino-nasal involvement in mucormycosis

Sinuses involved	*N*	%
Ethmoid	47	75.81
Maxillary	38	61.29
Sphenoid	32	51.61
Frontal	23	37.1
Pansinusitis	15	24.19

### MR imaging staging

MRI staging according to the extent of regional involvement. Stage 1 seen in 2 cases (3.23%), stage 2 in 13 cases (20.97%), stage 3 in 35 cases (56.45%) and stage 4 in 12 cases (19.35%), as detailed in Table 4.

**Table 4:** Staging according to the extent of regional involvement

Stage	Areas involved	*N* (%)
Stage 1	Limited to nasal mucosa	2 (3.23)
Stage 2	+ Paranasal sinuses	13 (20.97)
Stage 3	+ Orbit	35 (56.45)
Stage 4	+ Intracranial extension	12 (19.35)

### MR imaging characteristics of sino-nasal mucormycosis

Table 5 shows the pattern of sinus involvement, signal intensity, and enhancement characteristics on MR imaging.

**Table 5:** MR imaging pattern for sinus involvement

MR imaging characteristics	*N*	%
*Pattern of sinus infection*		
Sinus mucosal thickening	23	37.1
Complete sinus opacification	12	19.35
Combined pattern	27	43.55
*T1WI signal*		
Hypointense	62	100
*T2WI signal*		
Isointense/Hypointense	6	9.68
Hyperintense	23	37.1
Heterogeneous (high signal peripherally + low signal centrally)	33	53.23
*Sinus post-contrast enhancement*		
Absent	27	43.55
Present	35	56.45
Homogeneously mucosal sinus enhancement	7	11.29
Heterogeneously sinus enhancement	23	37.1
Rim enhancement with central non-enhancement	5	8.06
*Black turbinate sign*		
Absent	28	45.16
Present	34	54.84

### MRI analysis of extra-sinus extension

**Fig. 2:**
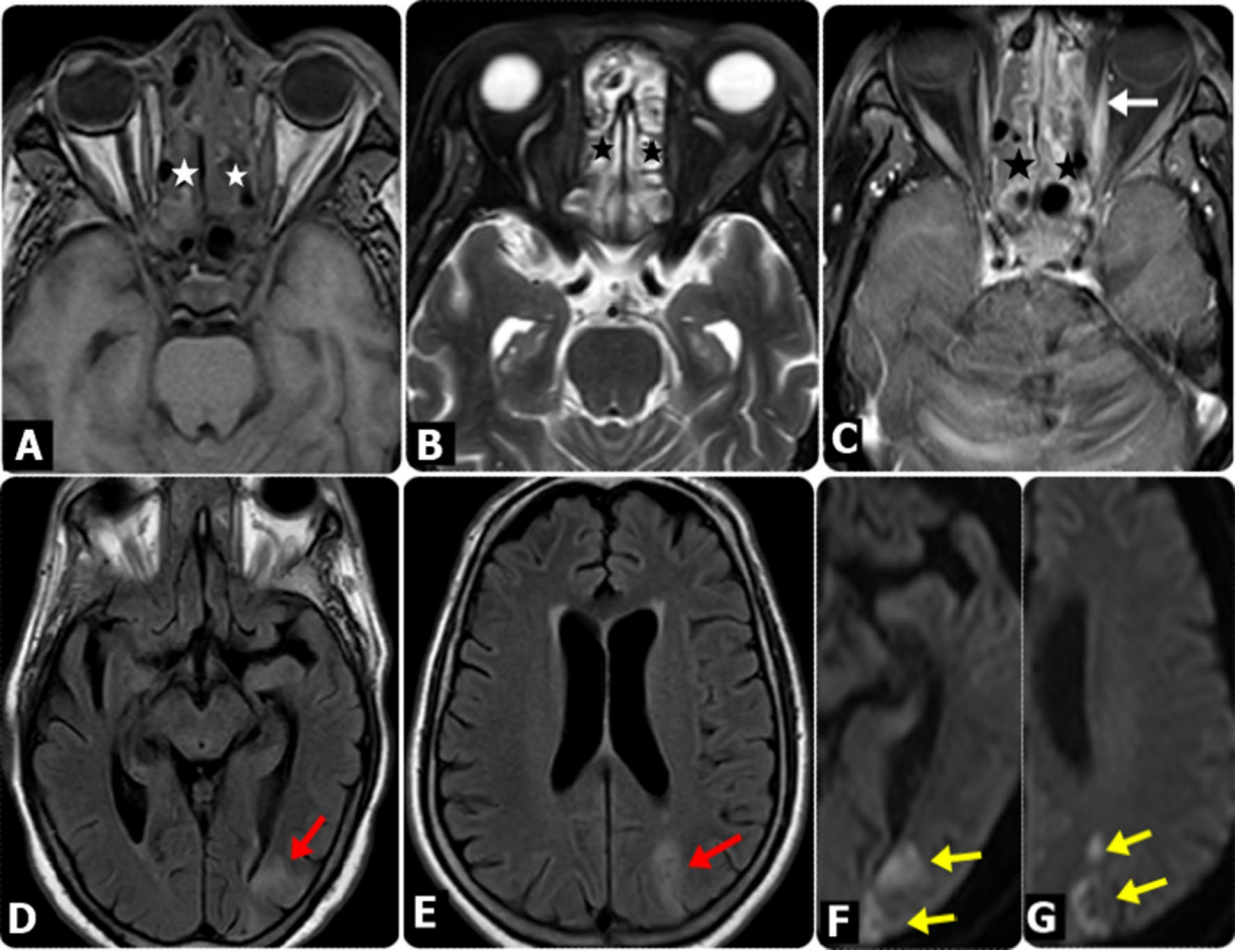
MRI findings in diabetic male patient, 55 years old, presented mainly with Lt. periocular and eyelids edema, following COVID-19. (**A**) Axial T1W, (**B**) and Axial T2 SPIR images showed bilateral ethmoidal sinusitis (Asterisks) and mild thickening of Lt. medial rectus muscle. **C** Axial post-contrast fat-saturated T1WI showed bilateral ethmoidal sinusitis with heterogeneous mucosal enhancement with mild thickened and enhanced Lt. medial rectus muscle (white arrows). **D** and **E** Axial FLAIR images; showed patchy hyperintense areas representing embolic acute infarcts at the Lt. occipital & posterior parietal regions (red arrows) which presented restricted diffusion on DW images (yellow arrows) at b-value 1000 (**F**) and (**G**)

**Fig. 3:**
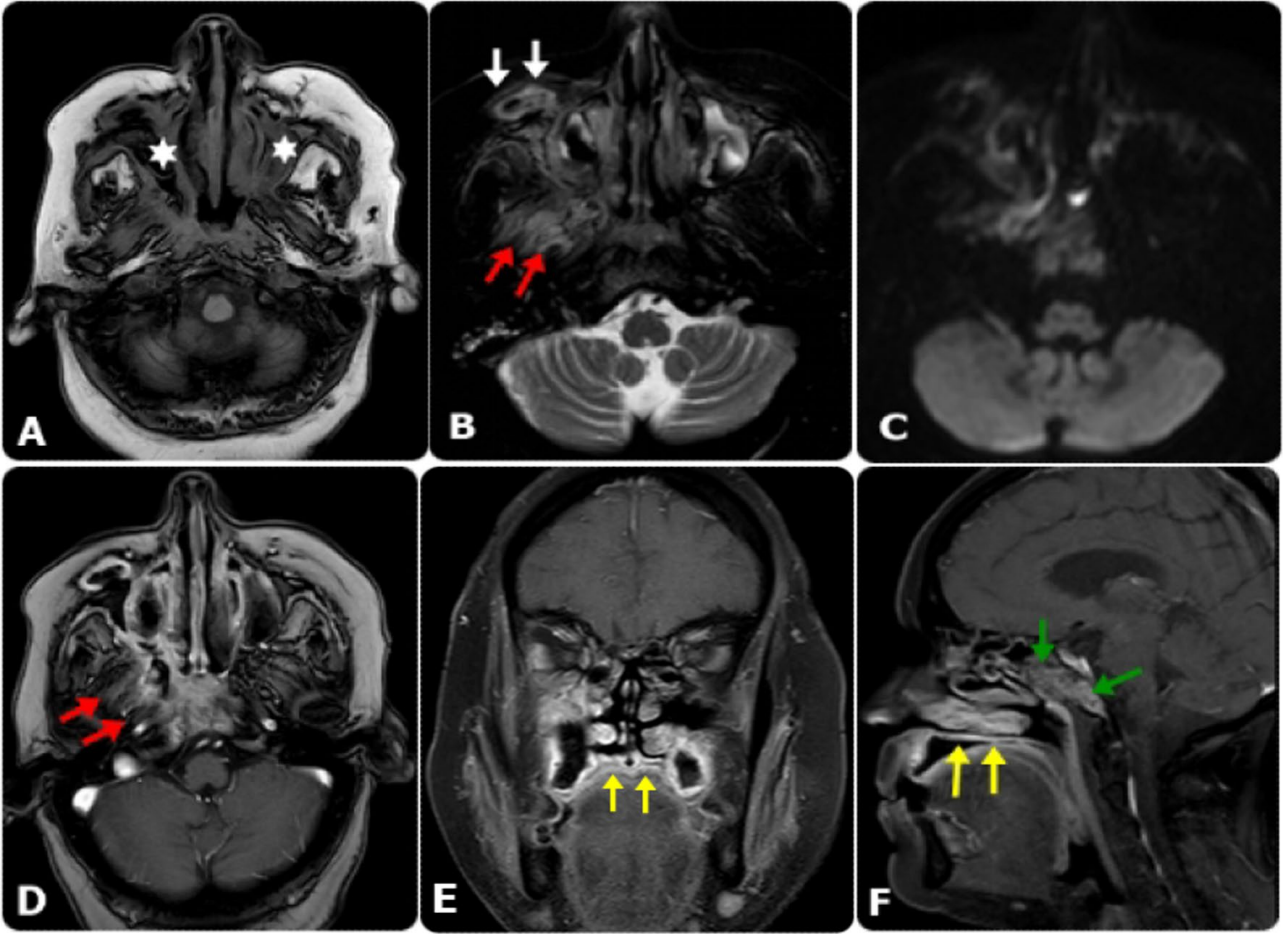
MRI findings in diabetic female patient, 56 years old, presented mainly with Rt. Maxillary swelling, following COVID-19. **A** Axial T1W, **B** and Axial T2-SPIR images showed bilateral maxillary sinusitis (Asterisks), with mixed signal intensity within representing heterogeneous pattern of affection, associated with detected Rt. Pre-maxillary small collection (white arrows), presented restricted diffusion on DWI at b-value 1000 (**C**) and showing peripheral pattern of enhancement on post-contrast axial T1W image (**D**). The edema and inflammatory process seen extending to the Rt. retro-maxillary region (Red arrows). Coronal (**E**) and sagittal (**F**) post-contrast fat-saturated T1W images revealed extension of the edema and inflammatory process to the hard palate (yellow arrows) and to the base of the skull (Green arrows)

**Fig. 4:**
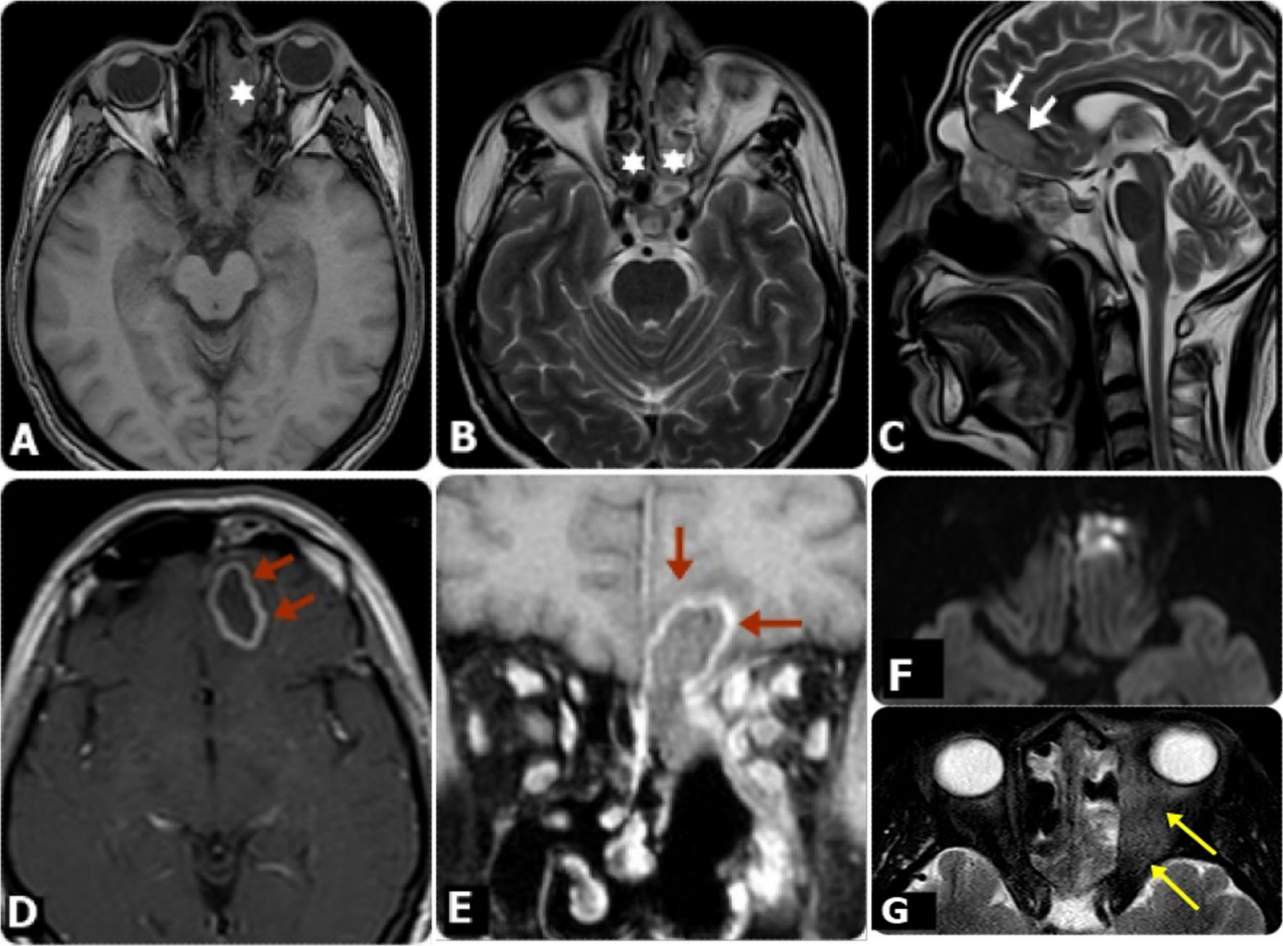
MRI findings in diabetic male patient, 59 years old, presented mainly with headache and altered mentation, in addition to Lt. eye proptosis, with periocular and eyelids edema, following COVID-19. **A** Axial T1W, **B** and Axial T2W images showed bilateral ethmoidal sinusitis (Asterisks), more evident at the Lt. side, with extension of the inflammatory process to the Lt. orbit. **C** Sagittal T2 WI, showed extension of the inflammatory process through the cribriform plate to the Lt. basi-frontal lobe (White arrows), exhibiting an area of high signal intensities, as well as frontal sinusitis. **D** Axial post-contrast T1WI and **E** coronal post-contrast fat-saturated T1WI showed marginally enhanced lesion at the Lt. basi-frontal lobe, which presented restricted diffusion on DW images at b-value 1000 (**F**), in keeping with cerebral abscess (Red arrows). Coronal post-contrast fat-saturated T1WI showed also extension of the inflammatory process to the Lt. orbit, resulting in mild enlargement of the related Lt. extra-ocular muscles. **G** Axial T2 SPIR image shows Lt. retro-ocular fat stranding (Yellow arrows)

**Fig. 5:**
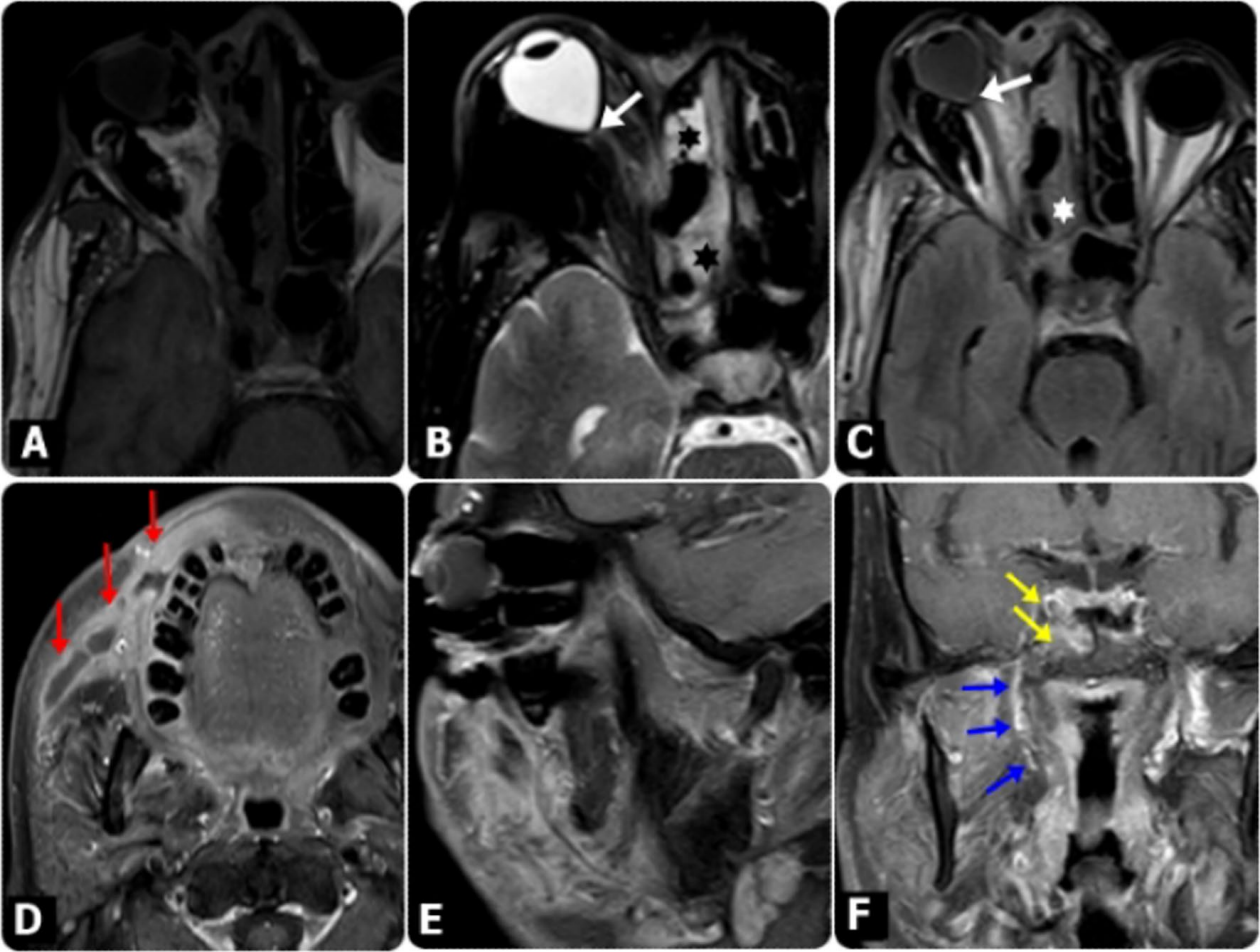
MRI findings in diabetic male patient, 49 years old, presented mainly with Rt. eye proptosis and Rt. premaxillary and periorbital edema, following COVID-19. **A** Axial T1W, **B** Axial T2-SPIR and **C** Axial FLAIR images showed ethmoidal sinusitis (Asterisks), more evident at the Rt. side, with extension of the inflammatory process to the Rt. orbit resulting in proptosis of Rt. eye globe, with posterior globe tenting (White arrows), as well as Rt. periorbital and eyelids edema. **D** Axial and **E** Sagittal post-contrast fat-saturated T1WI showed extension of the inflammatory process to the Rt. premaxillary region resulting in soft tissue edema and to the Rt. maxillary and zygomatic bones, showing heterogeneous post-contrast enhancement in keeping with bony invasion (red arrows). The edema and inflammatory process seen extending to the Rt. retro-maxillary region showing mild soft tissue contrast enhancement. **F** Coronal post-contrast fat-saturated T1WI showing mild enlarged right cavernous sinus with patchy heterogeneous enhancement (yellow arrows), with inflammatory spread to the Rt. infra-temporal fossa region (blue arrows)

**Fig. 6:**
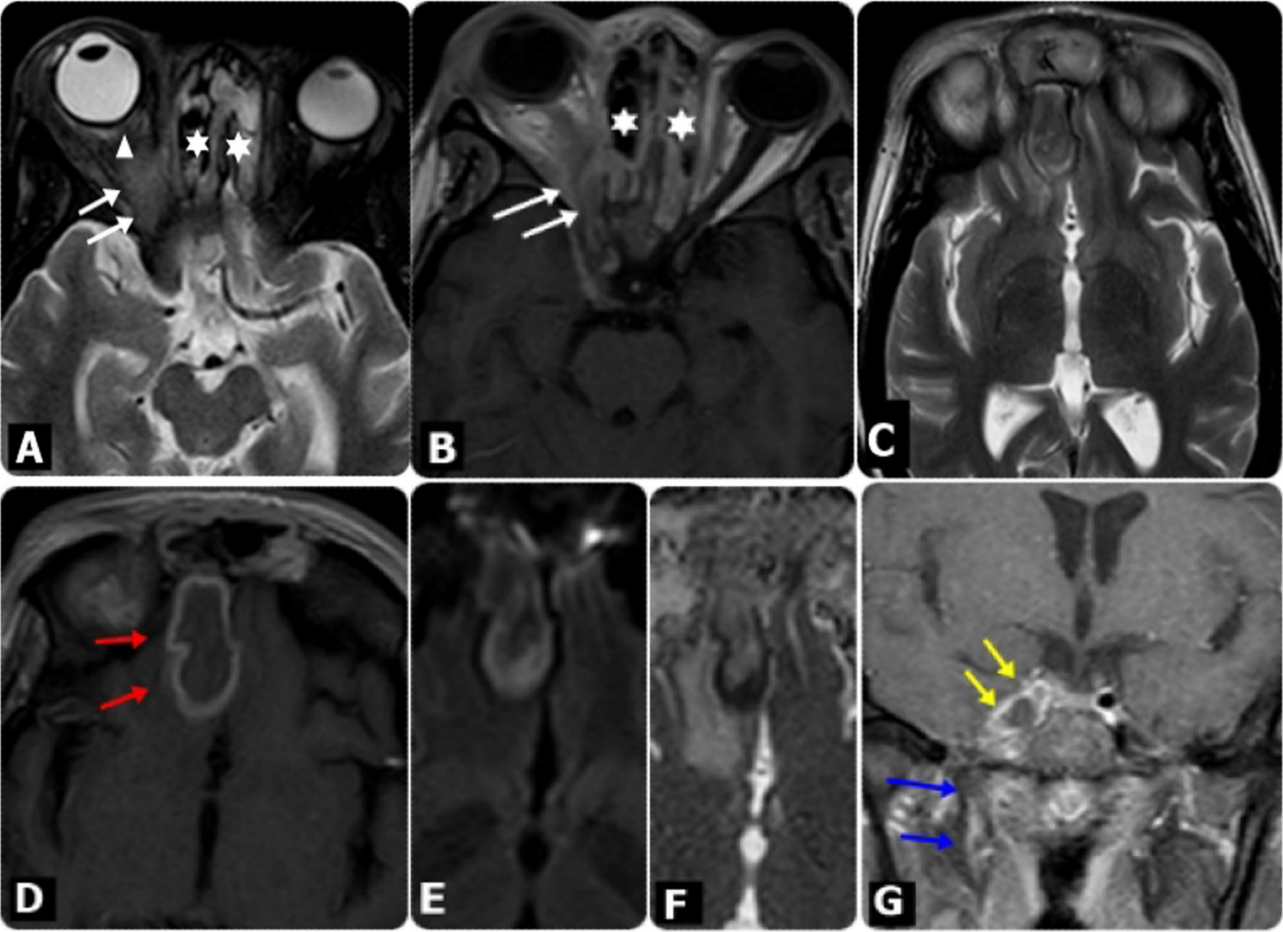
MRI findings in female patient, 36 years old, with chronic renal disease presented mainly with headache and altered mentation, in addition to Rt. eye proptosis, with periocular and eyelids edema, following COVID-19. **A** Axial T2 SPIR, **B** Axial post-contrast T1W images showed ethmoidal sinusitis (Asterisks), with extension of the inflammatory process to the Rt. orbit resulting in proptosis of Rt. eye globe, with posterior globe tenting (white arrow head), as well as Rt. periorbital and eyelids edema. There is retro-orbital fat stranding and heterogeneously enhancing soft tissue at the orbital apex (white arrows), that extend to the Rt. cavernous sinus. **C** Axial T2 WI showed extension of the inflammatory process to the Rt. basi-frontal lobe, exhibiting an area of abnormal signal intensities, that shows peripheral ring enhancement (red arrows) on axial post-contrast T1WI (**D**), presenting restricted diffusion on DW images at b-value 1000 (**E**) and apparent diffusion coefficient (ADC) map (**F**) in keeping with cerebral abscess. **G** Coronal post-contrast fat-saturated T1WI showing mild enlarged right cavernous sinus with patchy heterogeneous enhancement, with attenuated cavernous portion of the Rt. ICA (yellow arrows), associated with inflammatory spread to the Rt. infra-temporal fossa region (blue arrows)

**Fig. 7:**
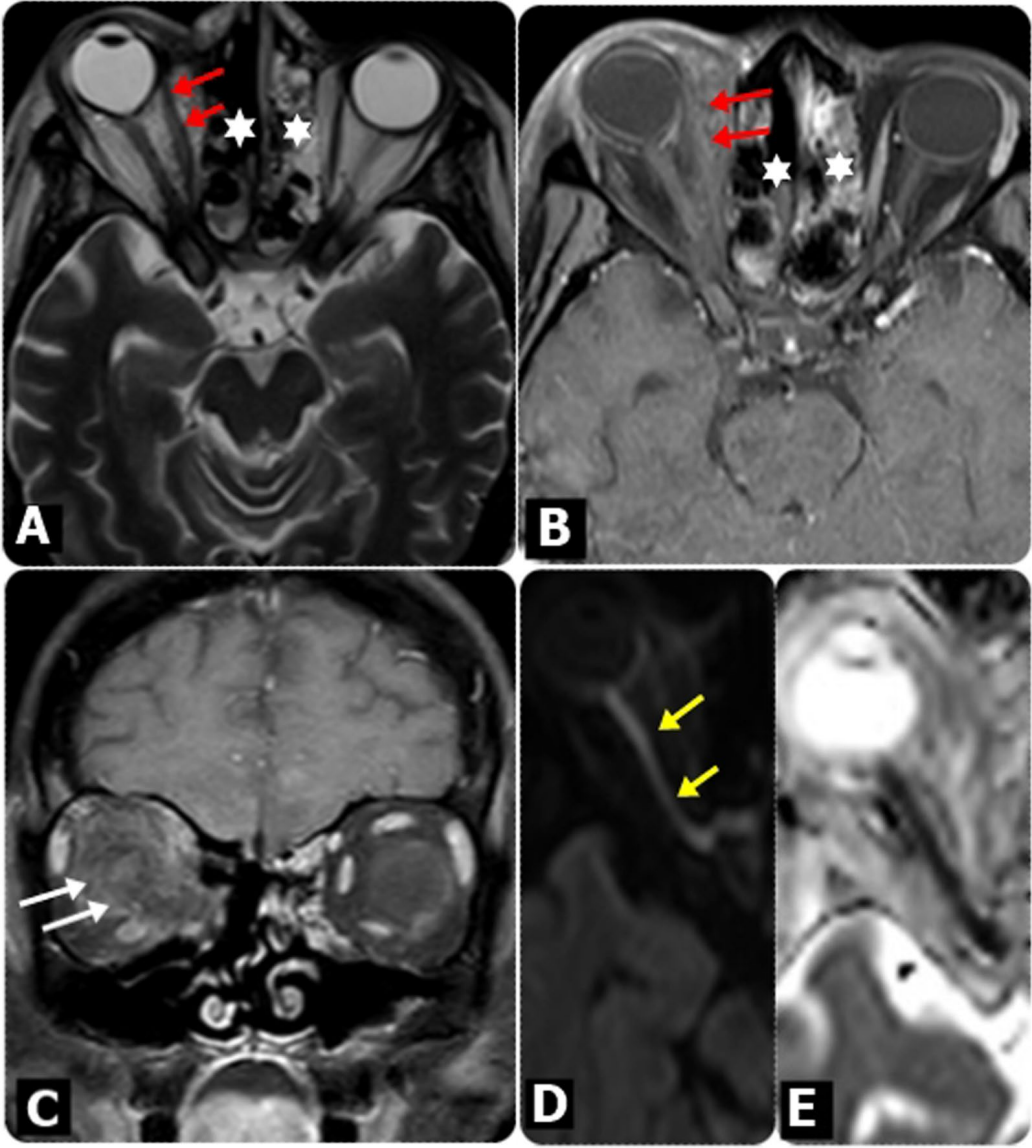
MRI findings in diabetic female patient, 65 years old, presented mainly with Rt. eye proptosis, with decreased in vision, following COVID-19. **A** Axial T2W as well as **B** Axial and **C** Coronal post-contrast fat-saturated T1WI showed bilateral ethmoidal sinusitis (asterisks), with extension of the inflammatory process to the Rt. orbit, resulting in proptosis of Rt. eye globe, with posterior globe tenting, as well as Rt. periorbital and eyelids edema. There is retro-orbital fat stranding (White arrows) and thickened and enhanced Rt. extra-ocular muscles (red arrows). Rt. optic nerve showed restricted diffusion on DW images (Yellow arrows) at b-value 1000 (**D**) and apparent diffusion coefficient (ADC) map (**E**) in keeping with Rt. optic nerve infarction

The sites and patterns of extra-sinus extension are summarized in Table 6. The most frequent site of extra-sinus extension was the pterygopalatine fossa (*n* = 47, 75.8%). Figures 2, 3, 4, 5, 6 and 7 show demonstrative cases.

**Table 6:** MR imaging pattern for extra sinus extension

Pattern of extra-sinus extension	*N* (%)	MRI features
*Maxilla-facial soft-tissue*		
Pre-antral soft tissue	43 (69.35)	Infiltration of the implicated soft-tissue spaces with fat stranding was primarily seen on T1 W, T2 W, fat-saturated T2W and postcontrast T1W images
Retro-antral fat	33 (53.23)	
Pterygopalatine fossa	47 (75.8)	
Infra-temporal fossa	22 (35.48)	
Pre-septal space	30 (48.39)	
Buccal space	5 (8.06)	
*Maxillo-facial bones*		
Anterior maxillary and zygomatic bone	2 (3.23)	Marrow edema and enhancement of the affected bones manifested primarily in fat-saturated T2W and postcontrast T1W images, respectively
Maxillary process of palatine bone	6 (9.68)	
*Orbit*		
Intraorbital intra-conal fat	12 (19.35)	T1W, fat-saturated T2W, and postcontrast T1W images showed retrobulbar fat stranding/edema and enhancing soft tissue in the orbit with or without extraocular muscle involvement
Intraorbital intra-conal soft tissue extension	8 (12.9)	
Including extra-ocular muscles	7 (11.29)	
Orbital apex	6 (9.68)	
Optic nerve	5 (8.06)	Optic nerve infarction is indicated by diffusion restriction within the nerve
Skull base	5 (8.06)	Bone marrow signal changes at the affected Clivus and pterygoid bones, along with marrow edema and enhancement, were most noticeable in fat-saturated T2W and postcontrast T1W images, respectively
*Intracranial*		
Cavernous sinus thrombosis	5 (8.06)	Abnormal signal intensity of the affected cavernous sinus, with abnormal enhancement, manifested mainly in fat-saturated T2W and postcontrast T1W images
ICA narrowing/occlusion	3 (4.84)	MR angiography and postcontrast T1W images demonstrated internal carotid artery constriction without or with arterial wall enhancement
Epidural/subdural collections	4 (6.45)	Epidural/sub-dural collections, with meningeal enhancement
Focal cerebritis	3 (4.84)	Cerebral parenchymal signal alterations with peripheral enhancement appreciated in T2W, diffusion-weighted and postcontrast T1W images indicating cerebral parenchymal invasion/abscess development
Cerebral infarction	3 (4.84)	Cortical and subcortical T2 and FLAIR hyperintense area with restricted diffusion following the vascular territory or in the watershed area indicating acute infarction

## Discussion

COVID-19 has been associated with a rise in the incidence of sino-nasal mucormycosis. We discussed the clinical manifestations and MR imaging findings for 62 individuals with sino-nasal mucormycosis in the context of COVID-19.

The present study showed a systematic approach for analyzing sino-nasal mucormycosis on MR imaging in post-COVID-19 patients, enabling a full evaluation of mucormycosis regional involvement and probable sites of extension. Furthermore, it demonstrated different disease imaging spectra.

Patients with post-COVID-19 sino-nasal mucormycosis were observed to have a median age of 50.65 ± 8.25 years, with 63% of them being female. Other research revealed a male-dominated population and a median age of over 55 years.

The most prevalent variant of mucormycosis infection is sino-nasal mucormycosis, which affect 50% of the patients [17]. Patients who are immunosuppressed and diabetic are more likely to develop it [6, 18].

Two meta-analyses found that DM was the primary risk factor for mucormycosis, representing 40% to 64% of cases [19, 20]. In our study, underlying diabetes mellitus as a predisposing factor was reported in 56/62 (90.3%) of mucormycosis cases and history of corticosteroid use was present in 48/62 (77.3%) patients.

According to reports, there can be a lag of 0 to 60 days between the diagnosis of COVID-19 and the onset of sino-nasal mucormycosis [9, 21], which is consistent with our finding that was found to be 25.7 (± 4.6) days.

In our study, the clinical presentation varied. The most common clinical symptoms were headache (52/83.87%) and the least was facial palsy seen in two patients.

Four main mechanisms proposed for the spread of sino-nasal mucormycosis are direct, perineural, perivascular, and hematogenous spread. The clinical manifestations and involved sites in each individual depend on the mode(s) of spread and the extent of involvement by the disease process [22]. Sixty-two (62) patients with mucormycosis had been included in our investigation. Patients initially exhibit sino-nasal involvement which may progress to the orbits, masticator space, face, pterygopalatine fossa, hard palate, maxillary alveolus, zygomatic process, skull base involving the Clivus and pterygoid process, and intracranial extension to involve the cavernous sinus, internal carotid artery, and cerebral hemispheres.

MRI provides more accurate assessment of soft tissue and intra-cranial involvement, invasion of the skull base, perineural dissemination, and vascular obstruction. Due to the iron and manganese in the fungal components, MRI shows varying signal intensity according on the sinus contents [23]. MRI contrast examination reveals invasion of the soft tissues of the orbit, infiltration of the skull base, perineural dissemination, intracranial complications and vascular obstruction involving internal carotid artery. T2 slow flow can predict internal carotid artery invasion by the fungus [24]. In head and neck cancers, particularly adenoid cystic carcinoma, perineural dissemination is seen frequently. Fungal hyphae frequently invade blood vessels and nerves, causing perineural spread and cavernous sinus invasion [25].

MR features of acute fulminant invasive fungal sinusitis (AFIFS) include black turbinate sign, (non-enhancing, hypointense turbinate), variable intensity within the sinuses on T1- and T2-weighted images (primary T2 low signals), obliteration of the nasopharyngeal planes, preantral fat infiltration, loss of contrast enhancement of the sino-nasal mucosa and extraocular muscles, inflammatory changes in the extraocular muscles and fat, and cerebral leptomeningeal enhancement [26, 27]. According to the results of the current study, in post-COVID-19 individuals, all of the previously recognized MRI mucormycosis characteristics were shown to varied degrees and did not differ from those seen in AFIFS.

Fungal infiltration or vascular congestion-related edema are the two main causes of extra-sinus fat infiltration, and as the fungus travels predominantly through perivascular channels, even before bone osteolysis occurs, it takes place [28, 29]. According to Gorovoy et al., distinct but late and less common features of AFIFS included retro-antral fat pad inflammation, osseous erosion, and orbital extension [28]. However, we reported the involvement of the retro-antral fat, facial bones, and orbit in 53.23% (33/62), 12.9% (8/62), and 56.45% (35/62) of our patients, respectively.

The orbit and brain tissues become infected with fungal growth due to direct vascular invasion or embolic seeding [28]. Additionally, Mathur et al. [30] discovered a link between the posterior ethmoid and sphenoid sinus being affected and a higher likelihood of intracranial extension [30].

In our investigation, we found no statistically significant correlation between extra-sinus extension to the orbit and brain and infiltration of the posterior ethmoid and sphenoid sinuses, as well as the maxillo-facial spaces.

The limitations of the present study include its small sample size, so a bigger multicenter cohort with a larger sample size is required to improve the accuracy of the results. Second, lack of an observed association of individual imaging findings with clinical outcomes. Finally, we did not demonstrate a difference in findings between sino-nasal mucormycosis in patients with COVID-19 and in other settings.

## Conclusions

Early diagnosis of sino-nasal mucormycosis and establishment of the scope of the infection depend heavily on MRI. As antifungal medications and surgical debridement can successfully manage the infection and hence lower the significant mortality and morbidity associated with mucormycosis, prompt diagnosis and treatment are the "sine qua non." It is crucial for all radiologists to be well-versed in the imaging characteristics of sino-nasal mucormycosis and its potential consequences given the evolving COVID-19 pandemic tendencies.

## Data Availability

The datasets used and/or analyzed during the current study are available from the corresponding author on reasonable request.

## Abbreviations

ADC: Apparent diffusion coefficient
AFIFS: Acute fulminant invasive fungal sinusitis
CE-MRI: Contrast enhanced magnetic resonance imaging
COVID-19: Corona virus disease of 2019
CT: Computed tomography
DM: Diabetes mellitus
DWI: Diffusion-weighted imaging
ENT: Ear, nose and throat
FLAIR: Fluid-attenuated inversion recovery
FS: Fat saturated
GRE: Gradient recalled echo
ICA: Internal carotid artery
KOH: Potassium hydroxide
MRI: Magnetic resonance imaging
ROCM: Rhino-orbitocerebral mucormycosis
RT-PCR: Reverse transcription-polymerase chain reaction
SARS-CoV-2: Severe acute respiratory syndrome corona virus-2
SD: Standard deviation
SPIR: Spectral Presaturation with Inversion Recovery
